# 

**Published:** 1876-10

**Authors:** 


### Table of Contents.

### ORIGINAL COMMUNICATIONS.

The Sulphate of Cinchonidia. *By Robert Battey, M.D., Rome, Ga.* 385

Oxygen Gas. *By G. B. Heard, M.D., LaGrange, Ga.* ........ 388

### REPORTS OF SOCIETIES.

Atlanta Academy of Medicine .......... 390

International Medical Congress .......... 393

### SELECTIONS.

Puerperal Scarlatina .......... 426

Death following the use of Ether .......... 435

### EDITORIAL.

A Manual of Percussion and Auscultation .......... 436

Chemistry .......... 436

Diseases of the Urinary Organs .......... 437

Diseases of the Eye .......... 437

Bloodletting in Purperal Eclampsia .......... 438

An Introduction to Pathology and Morbid Anatomy .......... 439

A Treatise on the Science and Practice of Midwifery .......... 439

Lectures on Fever .......... 439

The Pathology and Treatment of Childbed .......... 439

European Correspondence .......... 439

Salutatory .......... 446

The Scourge .......... 447

Valedictory .......... 448

